# Serum Metabolomic Alterations Associated with Cesium-137 Internal Emitter Delivered in Various Dose Rates

**DOI:** 10.3390/metabo10070270

**Published:** 2020-06-30

**Authors:** Heng-Hong Li, Yun-Tien Lin, Evagelia C. Laiakis, Maryam Goudarzi, Waylon Weber, Albert J. Fornace

**Affiliations:** 1 Department of Oncology, Georgetown University Medical Center, Washington, DC 20007, USA; yl737@georgetown.edu (Y.-T.L.); ecl28@georgetown.edu (E.C.L.); goudarm@ccf.org (M.G.); af294@georgetown.edu (A.J.F.J.); 2 Department of Biochemistry and Molecular & Cellular Biology, Georgetown University Medical Center, Washington, DC 20007, USA; 3 Lovelace Respiratory Research Institute, Lovelace Biomedical, Albuquerque, NM 87108, USA; wweber@lovelacebiomedical.org

**Keywords:** internal emitters, metabolomics, dose rate

## Abstract

Our laboratory and others have use radiation metabolomics to assess responses in order to develop biomarkers reflecting exposure and level of injury. To expand the types of exposure and compare to previously published results, metabolomic analysis has been carried out using serum samples from mice exposed to ^137^Cs internal emitters. Animals were injected intraperitoneally with ^137^CsCl solutions of varying radioactivity, and the absorbed doses were calculated. To determine the dose rate effect, serum samples were collected at 2, 3, 5, 7, and 14 days after injection. Based on the time for each group receiving the cumulative dose of 4 Gy, the dose rate for each group was determined. The dose rates analyzed were 0.16 Gy/day (low), 0.69 Gy/day (medium), and 1.25 Gy/day (high). The results indicated that at a cumulative dose of 4 Gy, the low dose rate group had the least number of statistically significantly differential spectral features. Some identified metabolites showed common changes for different dose rates. For example, significantly altered levels of oleamide and sphingosine 1-phosphate were seen in all three groups. On the other hand, the intensity of three amino acids, Isoleucine, Phenylalanine and Arginine, significantly decreased only in the medium dose rate group. These findings have the potential to be used in assessing the exposure and the biological effects of internal emitters.

## 1. Introduction

Nuclear plant disasters (Chernobyl, Fukushima-Daiichi) and the atomic bombs of Hiroshima and Nagasaki, in addition to extensive worldwide nuclear testing, released a high degree of radionuclides (e.g., ^137^Cs, ^90^Sr) in the surrounding areas. The fallout from these events, as well as future events, subsequently entered the food chain and water supplies, continuing the energy deposition and increasing the accumulated radiation doses received by a large number of individuals [1,2]. Such exposures may also be accidental [3] or intentional [4], as nuclear improvised devices and radiological dispersal devices remain elevated concerns for terrorist actions. ^137^Cs in particular is the most common fission product with a half-life of ~30 years, emitting γ radiation. Variable dose rates, generally below 1 Gy/day, depend on the amount of ingested radionuclides or groundshine [5] and have the potential to lead to variable effective doses and consequent biological effects, especially when external and internal exposures are combined [6], unless mitigated by administration of chelating drugs, such as Prussian blue [7].

Estimating the biological dose received from internal emitters is crucial in understanding how to properly assess exposure, medically treat affected populations, and understand the future risks of developing diseases such as cancer. However, it should be noted that the majority of the total dose received by an individual will be due to external exposure [8]. Cytogenetic analysis [9], the current gold standard for radiation assessment, remains laborious and time consuming, while newer methods, such as automated γ-H2AX foci counting and gene expression have shown potential in evaluating dose rate effects through internal emitters [10,11,12]. Recently, metabolomics has emerged as a method to determine biological dose and dose rate effects by assessing biofluids and developing biomarkers and biosignatures [13].

Metabolomics has been used extensively in developing radiation signatures for biodosimetry in biofluids in biological organisms ranging from murine models [14,15], to non-human primates [16,17], and total body irradiated patients [18,19]. Exposure to ionizing radiation induced significant changes of a variety of small molecules in mouse serum, including hypoxanthine, carnitine, proline, taurine, and some lipid mediators of inflammation. However, most of these studies have been focused on analyzing high dose rate data with external exposures. Limited studies have focused on internal exposures [20,21]. In this study we employed untargeted metabolomics through liquid chromatography time-of-flight mass spectrometry to characterize dose-rate effects from internal ^137^Cs exposure, with a cumulative dose of 4 Gy. Dose rate dependent and independent specific changes were identified, further demonstrating the potential of metabolomics to provide biomarkers of radiation exposure and the type of radiation.

## 2. Results

### 2.1. ^137^Cs Dose and DosE-Rate Calculation

**Figure 1:**
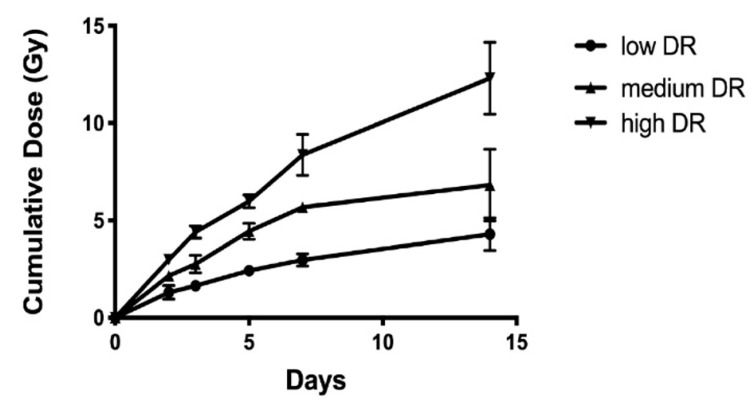
Calculated cumulative dose over the 14-day study period in three dose rate (DR) groups after administration of ^137^Cs with various activity.

The dose and dose-rate of intraperitoneally injected ^137^CsCl solutions were calculated according to the equations described in [22]. Mice received an injection of ^137^CsCl solutions in four groups with different activities ranging from 5.74 to 9.28 MBq. The cumulative total body dose and dose rate are summarized in Table 1 and Figure 1. As the animal number of the group receiving 6.66 MBq did not meet the experimental requirement of five or more for power analysis, this group was not included in the data analysis. Data of 4 Gy exposure with varying dose rates were compared to reveal the dose rate effects. Three groups with the administered activity of 5.74, 7.65 and 9.28 MBq reached a dose of 4 Gy on the fourteenth, the fifth, and the third day, respectively. The corresponding dose rates were 0.16 ± 0.05, 0.69 ± 0.05, and 1.25 ± 0.08 Gy/day for these three groups. In the following text, they will be referred to as low, medium, and high dose-rate, respectively.

### 2.2. Determine Differential Ions after Exposure to Internal Emitter

**Figure 2:**
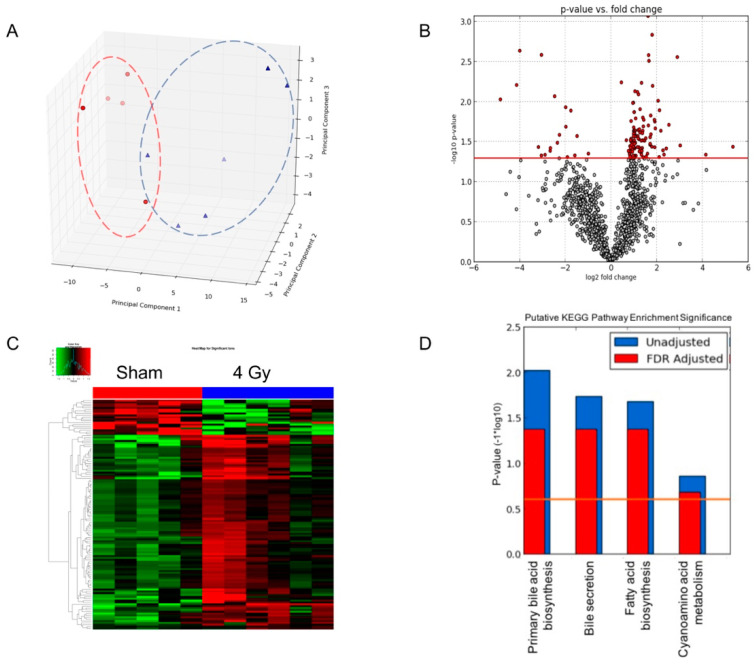
Comparative analysis of serum metabolomic profiles of control mice and those exposed to ^137^Cs at a cumulative dose of 4 Gy with high dose rate. (**A**) The principal component analysis (PCA) plot showing separation of metabolomic signatures from ^137^Cs-exposed (blue triangles) and control mice (red circles). (**B**) The volcano plot highlights statistically significant metabolites post-exposure (red dots). The *p*-value cutoff is 0.05. (**C**) The heatmap of metabolites whose levels changed significantly. (**D**) Molecular pathway enrichment analysis results of the differential ions identified in the multivariate analysis of serum metabolomic profiles.

The prepared serum samples were subjected to metabolomic profiling. The pairwise statistical analysis was performed for samples from mice that received the internal emitter compared with those from the sham control mice. 4 Gy exposure samples from each dose-rate group were compared with the corresponding sham controls. Figure 2 shows the results of statistical analysis on sera from mice that received high dose-rate of ^137^Cs exposure compared with the matched control. The PCA plot (Figure 2A) showed a clear separation between control and ^137^Cs-exposed mice. The volcano plot (Figure 2B) indicates the differential ions in ^137^Cs-exposed mice compared with controls. The ions with statistically significance (*p* < 0.05) are depicted as red dots. The intensity data of these differential ions are shown in the heatmap (Figure 2C). The putative identification of these differential ions was determined by screening the accurate mass in metabolite databases, as stated in the Methods section. KEGG pathway analysis results indicate the metabolic pathways associated with the differential metabolites. Prominent pathways with an FDR-corrected *p-*value less than 0.25 are shown in Figure 2D. The pairwise statistical analysis for low and medium dose-rate are shown in Figures S1 and S2. The PCA plot of sham and 4 Gy exposure for all three dose rate groups is shown in Figure S3.

### 2.3. DosE-Rate Effects on Metabolite Changes

**Figure 3:**
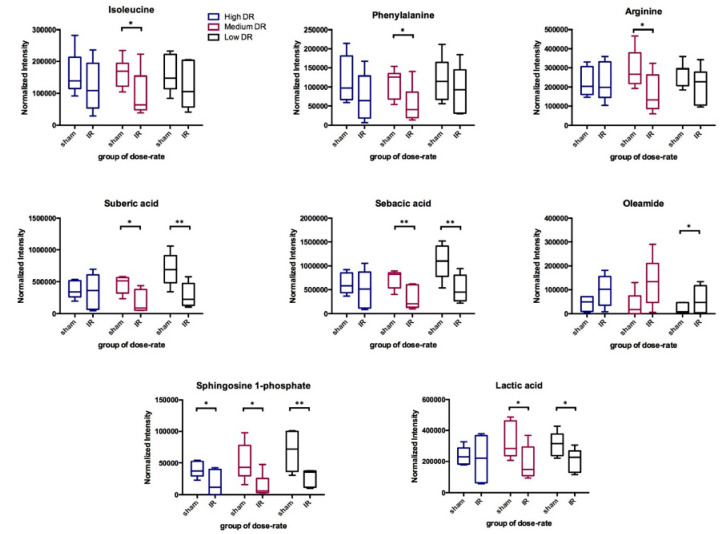
Changes in the serum levels of selected metabolites post ^137^Cs exposure. These metabolites were selected based on their statistical significance as determined by Mann-Whitney *U* test (value < 0.05) and biological importance. The identities of these ions were validated via MS/MS against pure standards and through MS/MS spectra published in METLIN databases. Data at the time points where a cumulative dose of 4 Gy achieved in each dose-rate group are showed.

**Figure 4:**
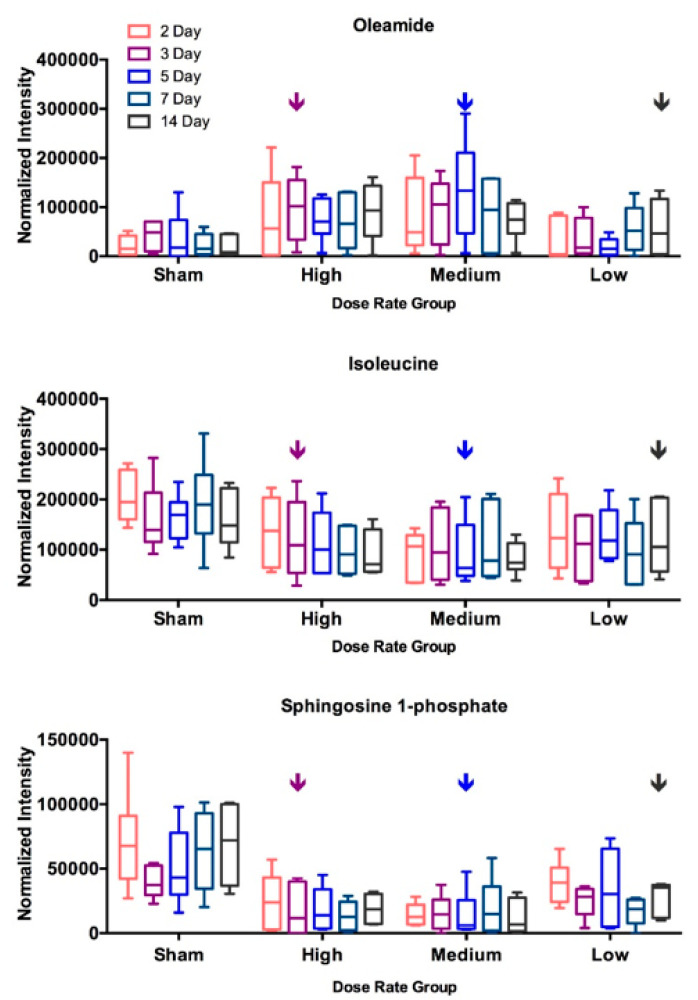
Various dose-rate effects are seen for different metabolites. The time-point pointed by the arrow in each dose-rate group indicates the time-point that achieved 4 Gy. The statistical significance of each post ^137^Cs exposure data point compared to its corresponding sham control are shown in Table 2.

Eight validated metabolites that showed significantly different levels after receiving 4 Gy in at least one dose rate condition are shown in Figure 3. Albeit significant differences were seen in only one or two dose rate conditions for most of these selected metabolites, similar trend of changes (i.e., either increase or decrease) was found in three dose rates tested. Data at five time points, from 2 days to 14 days after injection, of these metabolites were compared with the corresponding sham controls. The statistical significance for different time points is shown in Table 2. The cumulative dose increased along time points as shown in Table 1. The abundance ratio (4 Gy vs. sham) and its statistical significance of these selected metabolites are listed in the Table S1. Most of these eight metabolites showed significant changes in medium dose rate condition at one or more time points compared to sham. Isoleucine, phenylalanine, and arginine had significant decreases only in medium dose rate condition. Oleamide and lactic acid had significant changes at all three dose rates. At low and medium dose rates, significant changes were found at 4 Gy, however at the high dose rate condition, no significant changes were observed at 4 Gy but seen at higher doses, i.e., at 5 or more days after injection for these three metabolites. In contrast, sphingosine-1-phosphate (S1P) showed significant decreases in almost all time points at medium and high dose rates but not for time points less than 7 days at low dose rate. These data suggest that the metabolic changes associated with this internal emitter are related to the cumulative dose and dose rate received. Levels of three representative metabolites, oleamide, isoleucine, and sphingosine-1-phosphate, over the 14 days’ time course are shown in Figure 4.

## 3. Discussion

In the current study, a ^137^Cs injection model was used to study the dose rate effects of internal radiation emitter on serum metabolomics. The low dose rate in this study is 0.16 Gy/day, i.e., 6.67 mGy/h, close to the defined range of low dose rate ionizing radiation (LDRIR) of 6 mSv or less per hour [23]. While high dose rate radiation induced cell and tissue damage are well studied, the mechanisms of LDRIR-induced effects are poorly understood [24]. Metabolomic analysis comparing different dose rate effects were carried out after the cumulative dose reached 4 Gy. The low dose rate internal emitter induced a mild disturbance indicated by small number of differential metabolites upon exposure compared to that from animals receiving similar dose delivered with medium and high dose rates.

S1P is an important bioactive lipid mediator, associated with various stress conditions and diseases [25,26,27]. S1P and its precursor, ceramide, are reciprocal regulators of cellular fate. Ceramide-mediated apoptosis is a well characterized cell death mechanism after radiation exposure [28,29]. S1P, a ceramide antagonist, has been known for acting as a protectant of radiation-induced apoptosis [30,31]. In our study, the serum level of S1P decreased after radiation exposure in most of the dose and dose rate conditions, except in the groups that received less than 2.97 ± 0.31 Gy of cumulative dose with low dose rate. However, at the time points when the equivalent cumulative dose reached in medium and high dose rate groups, significant decreases of S1P were observed as shown in Table 2. Our results indicate that the change of serum S1P level showed a dose-dependent manner with a threshold for internal emitter at low dose rate, but not for the exposure of medium and high dose rates.

## 4. Materials and Methods

### 4.1. Animal Irradiation and Sample Collection

Ten- to twelve-week-old male wild type C57Bl/6 mice purchased from Charles River Laboratories (Frederick, MD) were quarantined for 14 days prior to random group assignment into the study. Animals were administered ^137^CsCl solutions in a volume of 50 μL with varying activity from 5.74 to 9.28 MBq through intraperitoneal injection. Mice were housed individually after injection in microisolator cages with lead shielding used to minimize cross-irradiation from adjacent mice. Mice were sacrificed at 2, 3, 5, 7, and 14 days after injection and serum was collected at necropsy by cardiac puncture. Sham-irradiated mice were included in each dose rate group, sacrificed at the corresponding time points. Animal studies were approved by the Lovelace Respiratory Research Institute (LRRI) Institutional Animal Care and Use Committee under protocol number FY15-087, in facilities accredited by the Association for Assessment and Accreditation of Laboratory Animal Care International; and carried out in compliance with the Guide for the Care and Use of Laboratory Animals (National Research Council 2011).

### 4.2. Sample Preparation for Mass Spectrometry Analysis

25 μL serum was added to tubes followed by 100 μL cold chloroform/methanol (2/1) as described previously [32]. The mixture was vortexed for 30s at room temperature and then centrifuged at 13,000× *g* for 5 min to separate the polar and nonpolar species. The upper phase containing polar metabolites was collected and transferred to new tubes. The white protein interphase was discarded. The collected samples were dried using a speed vacuum. The pellet was suspended in 100 μL of 50% acetonitrile.

### 4.3. Mass Spectrometry Analysis

The chromatographic and mass spectrometric parameters used were described previously [32]. The Ultra Performance Liquid Chromatography (UPLC) column eluent was introduced directly into the mass spectrometer by electrospray. For metabolomic profiling, 2 μL sample was injected into a reverse-phase 50 × 2.1 mm H-class UPLC Acquity 1.7-μM BEH C18 column (Waters Corp., Milford, MA, USA) coupled to a time-of-flight mass spectrometry system (TOFMS). Mass spectrometric analysis was performed on a XEVO G2 QTOF (Waters Corp., Milford, MA, USA) operating in both positive and negative ionization modes. Accurate mass was maintained by introduction of LockSpray interface of sulfadimethoxine (311.0814 [M+H]^+^ or 309.0658 [M-H]^−^) at a concentration of 250 pg/μL in 50% aqueous acetonitrile and a rate of 150 μL/min. All chromatograms and mass spectrometric data were acquired in centroid mode using the MassLynx software (Waters Corp., version 4.1, Milford, MA, USA).

### 4.4. Data Processing and Multivariate Data Analysis

Raw mass spectrometric data were processed using Progenesis QI software (Nonlinear Dynamics, version 2.0, Durham, NC, USA) to generate a data matrix that consisted of the retention time, *m/z* value, and the normalized abundance using the default method, normalise to all compounds. Statistical analysis and putative ion identification on the postprocessed data were conducted using MetaboLyzer as described previously [33], which utilizes the Human Metabolome Database (HMDB), LipidMaps, and the Kyoto Encyclopedia of Genes and Genomes (KEGG) database while accounting for possible adducts, H^+^, Na^+^, and NH_4_^+^ in the ESI^+^ mode, and H^−^ and Cl^−^ in the ESI^−^ mode. Data were log transformed. To identify differential ions, Mann-Whitney *U* test was performed for ions with >70.0% presence in the dataset. Barnard’s test was used for the rest of the ions that did not reach the ion presence percentage cutoff. False discovery rate (FDR) based multiple testing correction was performed with FDR of 0.2. Principal component analysis (PCA) was performed to provide three-dimensional visualization of the data. The *m/z* values were compared with the exact mass of small molecules in the databases, from which putative metabolites were identified with a mass error of 10 ppm or less. KEGG annotated pathways associated with these putative metabolites were also identified. Validation of selected putative metabolites was done with tandem mass spectrometry (MS/MS), fragmentation patterns were compared with those of the pure chemicals and cross-referenced to MS/MS spectra in the METLIN library. Principal components analysis (PCA) shown in Figure S3 was performed on Pareto-scaled data using SIMCA-P+ 12.0 software (Umetrics, version 12.0, Kinnelon, NJ, USA).

### 4.5. Statistics

Statistically significance was based on the results from MetaboLyzer with the Mann-Whitney *U* test and Barnard’s test. The box and whisker plots of metabolite intensity data were generated using GraphPad Prism 6 Software (GraphPad Software, version 6, San Diego, CA, USA).

## 5. Conclusions

The previous work by our laboratory studied serum metabolites changes after receiving different doses at various times of internal exposure by a single dose of ^137^CsCl [20,21]. While fatty acids and phosphatidylcholines were identified as the most perturbed ions in the serum of ^137^Cs-exposed mice by a lipidomics method, the previous study also showed modest changes of amino acids and metabolites associated with glycolysis. This study focuses on internal exposure with different doses and dose rates, and indicates minimal changes on the serum metabolome after internal emitter exposure delivered at low dose rate. Some metabolites showed significant changes within a certain range of dose rate and others can have universal changes with mild effect of dose rate. These findings have the potential to be used in assessing the exposure and the biological effects of internal emitter.

## Figures and Tables

**Table 1:** Total-body dose (Gy) and dose rates (Gy/day) measured over the 14-day study period. The dose and the corresponding dose rates on the day when the accrued dose reached 4 Gy are marked in red for each group.

Dose Rate Group	Time Point (d)	WholE-body Committed Dose (Gy)	Dose Rates (Gy/day)	Number of Samples
**Sham**	2	0.00	0.00	6
	3	0.00	0.00	6
	5	0.00	0.00	6
	7	0.00	0.00	6
	14	0.00	0.00	8
**Low Dose Rate**	2	1.31 ± 0.36	0.61 ± 0.17	6
	3	1.65 ± 0.07	0.47 ± 0.02	6
	5	2.42 ± 0.14	0.39 ± 0.03	6
	7	2.97 ± 0.31	0.3 ± 0.04	6
	14	4.3 ± 0.84	0.16 ± 0.05	7
**Medium Dose Rate**	2	2.16 ± 0.17	0.97 ± 0.08	5
	3	2.76 ± 0.45	0.78 ± 0.11	6
	5	4.44 ± 0.41	0.69 ± 0.07	6
	7	5.68 ± 0.24	0.58 ± 0.03	6
	14	6.81 ± 1.84	0.24 ± 0.11	6
**High Dose Rate**	2	2.98 ± 0.14	1.36 ± 0.07	6
	3	4.41 ± 0.32	1.25 ± 0.08	6
	5	5.98 ± 0.32	0.94 ± 0.05	6
	7	8.37 ± 1.05	0.85 ± 0.08	6
	14	12.31 ± 1.86	0.85 ± 0.08	6

**Table 2:** Dose response in different dose-rate groups indicated by statistical difference of these validated ions. *, *p*-value < 0.05; **, *p*-value < 0.01.

Dose Rate	Low (0.16 Gy/day)					Medium (0.69 Gy/day)					High (1.25 Gy/day)				
	2 Day	3 Day	5 Day	7 Day	14 Day	2 Day	3 Day	5 Day	7 Day	14 Day	2 Day	3 Day	5 Day	7 Day	14 Day
Suberic Acid				*	*			*		*					*
Sebacic Acid					*			**		*					*
Lactic Acid					*			*	*	*			*	**	*
L-Isoleucine						*		*		*					
L-Phenylalanine						*		*							
L-Arginine						*		*		*				*	*
Oleamide					*								*		*
Sphingosine 1-phosphate				*	**	**	*	*	**	**	*	*		*	**
Uric Acid								**	*				*	*	

## Supplementary Materials

The following are available online at https://www.mdpi.com/2218-1989/10/7/270/s1, Figure S1: Comparative analysis of serum metabolomic profiles of control mice and those exposed to ^137^Cs at a cumulative dose of 4 Gy with low dose rate, Figure S2: Comparative analysis of serum metabolomic profiles of control mice and those exposed to ^137^Cs at a cumulative dose of 4 Gy with medium dose rate, Figure S3: The PCA plot of serum metabolomic profiles of sham control mice and those exposed to ^137^Cs at a cumulative dose of 4 Gy, Table S1: The abundance ratio (4Gy vs. sham) for various dose rates and the corresponding *p*-value of selected differential metabolites.
