# Supplementary material for: Serum Metabolomic Alterations Associated with Cesium-137 Internal Emitter Delivered in Various Dose Rates
Source: Metabolites. 2020 Jun 30;10(7):270. doi: 10.3390/metabo10070270 (PMC7407308; doi:10.3390/metabo10070270)
Supplement: Supplementary file 1 [file metabolites-10-00270-s001.pdf]

Supplementary file

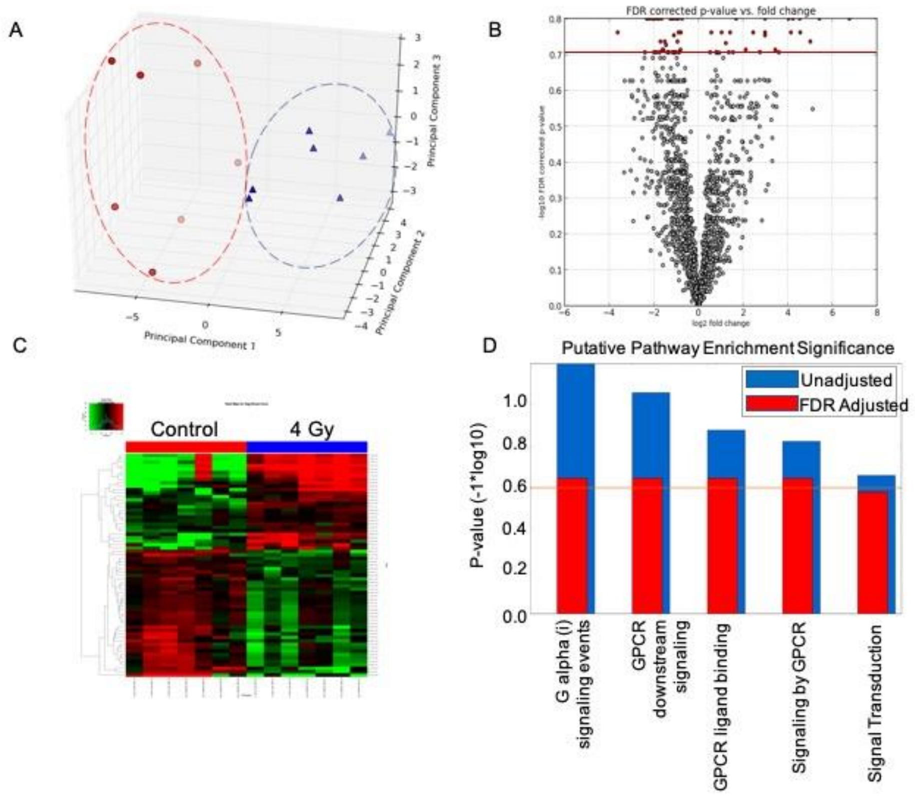

SFig.1. Comparative analysis of serum metabolomic profiles of control mice and those exposed to ^{137}Cs at a cumulative dose of 4 Gy with low dose rate. (A) The PCA plot showing separation of metabolomic signatures from ^{137}Cs -exposed (blue triangles) and control mice (red circles). (B) The volcano plot highlights statistically significant metabolites post-exposure (red dots). (C) The heatmap of metabolites whose levels change significantly. (D) Molecular pathway enrichment analysis results of the differential ions identified in the multivariate analysis of serum metabolomic profiles.

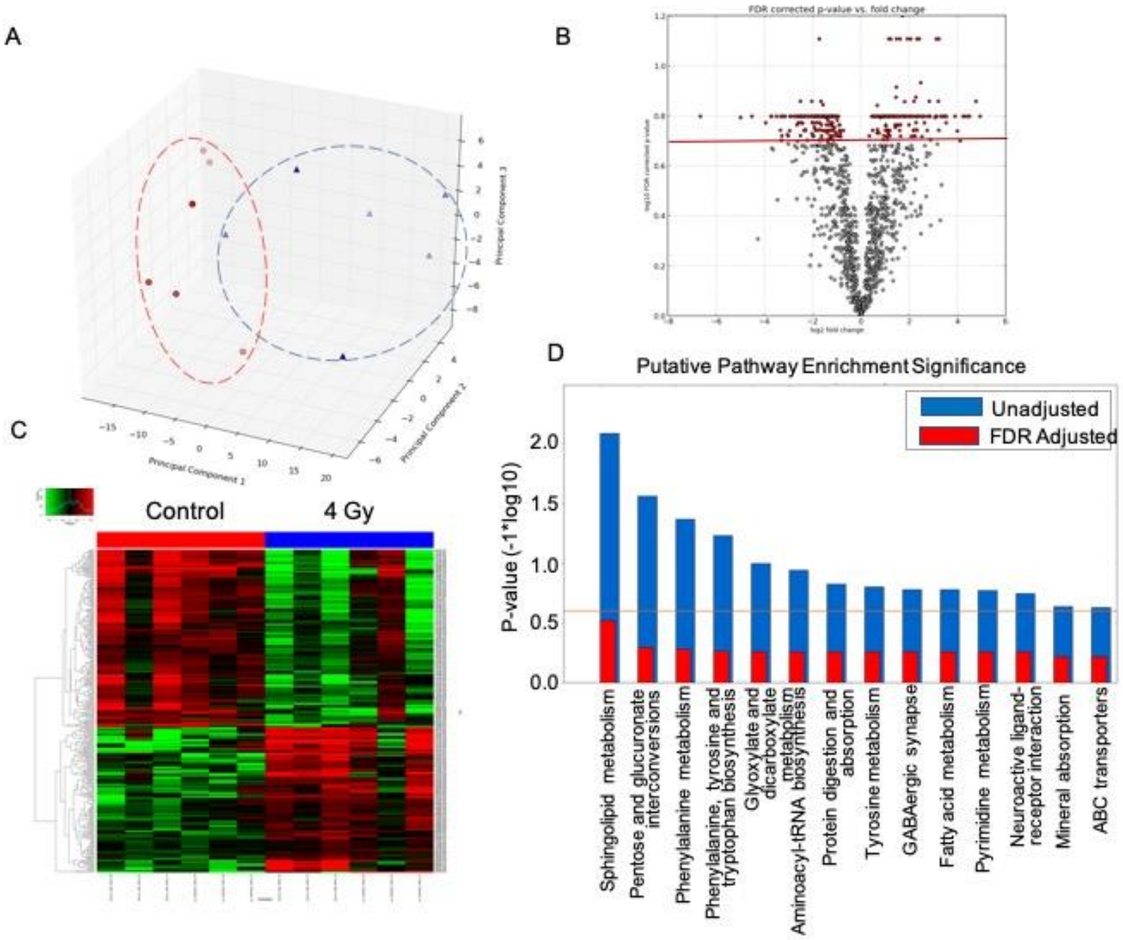

SFig.2. Comparative analysis of serum metabolomic profiles of control mice and those exposed to ^{137}Cs at a cumulative dose of 4 Gy with medium dose rate. (A) The PCA plot showing separation of metabolomic signatures from ^{137}Cs -exposed (blue triangles) and control mice (red circles). (B) The volcano plot highlights statistically significant metabolites post-exposure (red dots). (C) The heatmap of metabolites whose levels change significantly. (D) Molecular pathway enrichment analysis results of the differential ions identified in the multivariate analysis of serum metabolomic profiles.

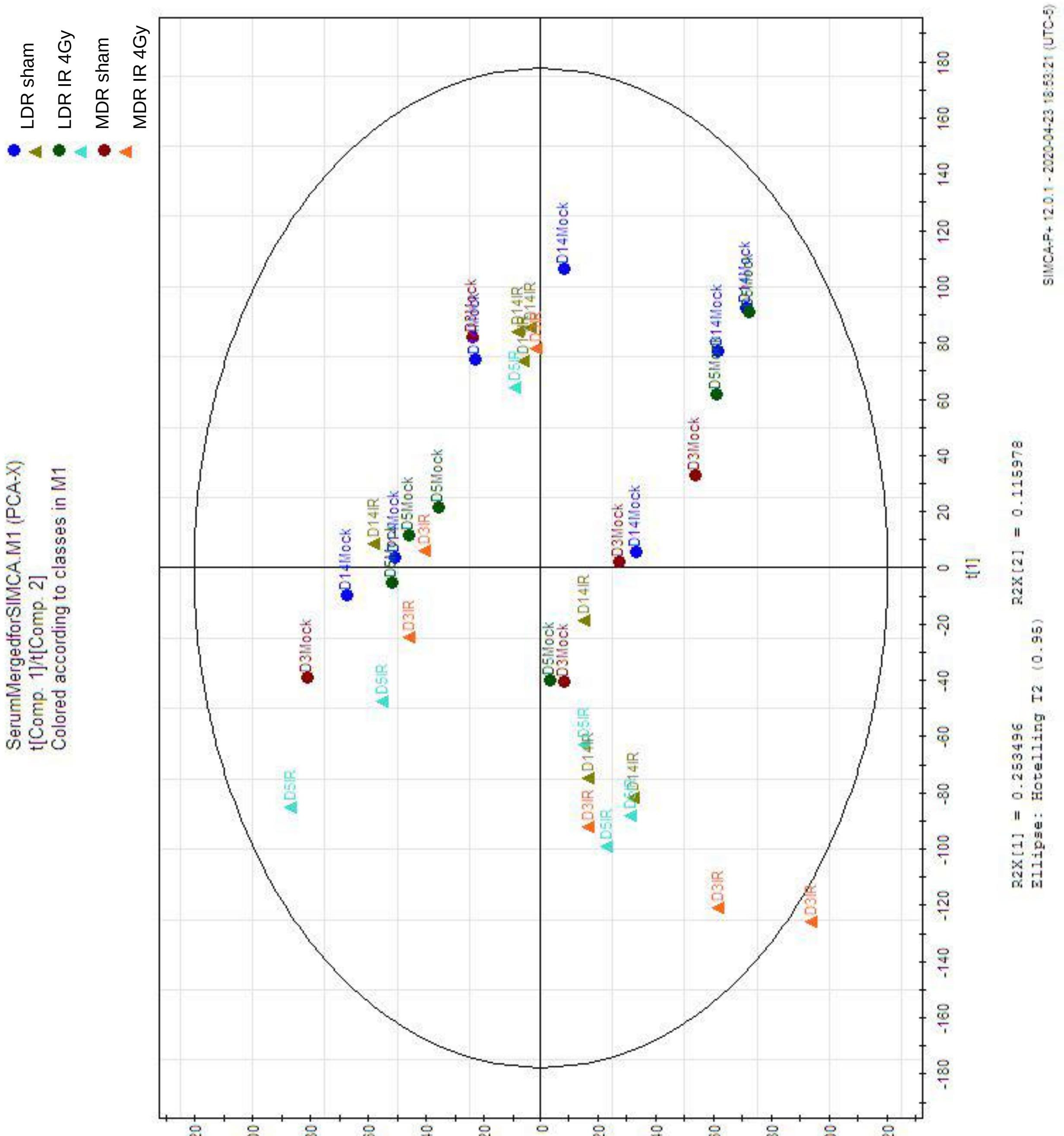

SFig.3. The PCA plot of serum metabolomic profiles of sham control mice and those exposed to ^{137}Cs at a cumulative dose of 4 Gy.

Supplementary Table 1. The abundance ratio (4Gy vs. sham) for various dose rates and the corresponding p-value of selected differential metabolites.

Metabolite	low dose rate		medium dose rate		high dose rate	
	Ratio (4Gy/sham)	p-value	Ratio (4Gy/sham)	p-value	Ratio (4Gy/sham)	p-value
Suberic Acid	0.47	0.01	0.39	0.012	0.77	0.508
Sebacic Acid	0.51	0.01	0.43	0.008	0.71	0.345
Lactic Acid	0.66	0.018	0.58	0.050	0.75	0.412
Arginine	0.84	0.368	0.58	0.048	0.89	0.620
Isoleucine	0.78	0.312	0.58	0.048	0.75	0.363
Phenylalanine	0.84	0.563	0.49	0.046	0.56	0.144
Sphingosine-1-P	0.41	0.01	0.27	0.029	0.45	0.043
Oleamide	1.88	0.048	3.64	0.067	2.29	0.099
Uric acid	0.62	0.087	0.32	0.011	0.69	0.074
